# Ethanol production from dilute‐acid steam exploded lignocellulosic feedstocks using an isolated multistress‐tolerant *Pichia kudriavzevii* strain

**DOI:** 10.1111/1751-7915.12712

**Published:** 2017-05-05

**Authors:** Shuo‐Fu Yuan, Gia‐Luen Guo, Wen‐Song Hwang

**Affiliations:** ^1^ Institute for Cellular and Molecular Biology The University of Texas at Austin Austin TX USA; ^2^ Chemistry Division Institute of Nuclear Energy Research Atomic Energy Committee Executive Yuan, No. 1000 Wenhua Rd. Jiaan Village, Longtan District Taoyuan City 32546 Taiwan

## Abstract

Renewable and low‐cost lignocellulosic wastes have attractive applications in bioethanol production. The yeast *Saccharomyces cerevisiae* is the most widely used ethanol‐producing microbe; however, its fermentation temperature (30–35°C) is not optimum (40–50°C) for enzymatic hydrolysis in the simultaneous saccharification and fermentation (SSF) process. In this study, we successfully performed an SSF process at 42°C from a high solid loading of 20% (w/v) acid‐impregnated steam explosion (AISE)‐treated rice straw with low inhibitor concentrations (furfural 0.19 g l^−1^ and acetic acid 0.95 g l^−1^) using an isolate *Pichia kudriavzevii* SI, where the ethanol titre obtained (33.4 g_p_ l^−1^) was nearly 39% greater than that produced by conventional *S. cerevisiae* BCRC20270 at 30°C (24.1 g_p_ l^−1^). In addition, *P. kudriavzevii* SI exhibited a high conversion efficiency of > 91% from enzyme‐saccharified hydrolysates of AISE‐treated plywood chips and sugarcane bagasse, although high concentrations of furaldehydes, such as furfural 1.07–1.21 g l^−1^, 5‐hydroxymethyl furfural 0.20−0.72 g l^−1^ and acetic acid 4.80–7.65 g l^−1^, were present. This is the first report of ethanol fermentation by *P. kudriavzevii* using various acid‐treated lignocellulosic feedstocks without detoxification or added nutrients. The multistress‐tolerant strain SI has greater potential than the conventional *S*.* cerevisiae* for use in the cellulosic ethanol industry.

## Introduction

Because of the high price of petroleum and environmental concerns, the necessity for renewable fuel is growing rapidly. Lignocellulosic biomass is a readily available, abundant and renewable source of energy. Approximately 5.6 billion dry tons of plant biomass‐derived agricultural and forestry residues are produced annually, although only 10–25% of them could be used for producing biofuel (Ho *et al*., 2014).

Lignocellulosic biomass is predominantly composed of lignin and carbohydrate polymers (cellulose and hemicellulose). The presence of lignin and hemicellulose in lignocelluloses forms a recalcitrant barrier around cellulose, which protects plant cells from destruction by fungi or bacteria (Zhao *et al*., 2012).One of the critical steps in the conversion of biomass into fuel ethanol is the hydrolysis of hemicellulose and cellulose to yield monomeric sugars, which can be further utilized by fermenting microorganisms. However, the sugar compositions and amounts vary with the lignocellulosic feedstock and the specific saccharification technology employed (Sarkar *et al*., 2012).To break down the physicochemical structure of lignocellulose in an efficient manner, various pretreatment strategies, including dilute‐acid hydrolysis, steam explosion, liquid hot water extraction and alkaline hydrolysis, have been developed and used prior to the enzymatic hydrolysis and ethanol fermentation steps (Kumar *et al*., 2009). Among these pretreatments, dilute‐acid hydrolysis and steam explosion have been tested widely in pilot‐scale equipment and are considered favourable methods for industrial applications (Wyman *et al*., 2005; Taherzadeh, 2007; Modenbach and Nokes, 2012). However, various lignocellulose‐derived inhibitors such as weak acids, furan derivatives and phenolic compounds, which greatly inhibit microbial growth, are generated by the dehydration of hexoses and pentoses during the acid hydrolysis process (Palmqvist and Hahn‐Hägerdal, 2000a). Another crucial factor that greatly affects the cost of ethanol production is the temperature used for fermentation in large‐scale systems (Abdel‐Banat *et al*., 2010). Fermentation at high temperatures is advantageous for reducing the energy input, increasing the efficiency of product recovery and decreasing the possibility of contamination (Hasunuma and Kondo, 2012). Therefore, thermotolerant microbes with efficient ethanol production and high tolerance of multiple stressors are desirable for use in the bioethanol industry.

Several thermotolerant yeasts have been characterized, and *Pichia kudriavzevii*, known as *Issatchenkia orientalis* (Kurtzman *et al*., 1980), has been recently shown to produce significantly larger amounts of ethanol in the presence of high salt concentrations in an acidic medium or at high incubation temperatures than the high ethanol‐producing yeast *Saccharomyces cerevisiae* (Isono *et al*., 2012). Previous studies have reported the application of *P. kudriavzevii* to ethanol production from cassava starch hydrolysate, alkali‐treated rice straw, alkali‐ and ozone‐treated cotton stalks and sugarcane juice at high temperatures (Dhaliwal *et al*., 2011; Kaur *et al*., 2012; Oberoi *et al*., 2012; Yuangsaard *et al*., 2013). However, no previous studies have reported the use of inhibitor‐tolerant *P. kudriavzevii* for ethanol production from acid‐treated lignocellulosic biomass without detoxification at high temperatures.

In this study, we isolated and characterized thermotolerant yeast, *P. kudriavzevii* SI, and we compared the effects of lignocellulose‐derived inhibitors on ethanol fermentation using *P. kudriavzevii* SI and conventional *S. cerevisiae* BCRC20270. Moreover, we performed non‐detoxified fermentation from dilute‐acid‐ and steam explosion‐pretreated lignocellulosic materials, including rice straw, plywood chips and sugarcane bagasse, by simultaneous saccharification and fermentation (SSF) and by separate hydrolysis and fermentation (SHF) to demonstrate the potential of this inhibitor‐tolerant strain for use in cost‐effective ethanol production at high temperatures.

## Results and discussion

### Isolation and identification of thermotolerant yeast strains

Yeast strains that tolerated high temperature were isolated from kitchen waste at 40°C using yeast–peptone–dextrose(YPD) medium. The composition of kitchen waste that yeast strain was isolated contained nearly 90% liquid with 4.9 ± 0.4 of pH, 36.9 ± 20.5 g l^−1^ of sugar (fructose and glucose), 5.7 ± 1.0 g l^−1^ of lactic acid, 2.4 ± 0.3 g l^−1^ of acetic acid and 6.8 ± 0.9 g l^−1^ of ethanol. This screening process identified a strain that produced 10.2 g l^−1^ of ethanol from 21.9 g l^−1^ of glucose, which corresponded to a 91.3% of the theoretical yield and produced 0.22 g l^−1^ of acetic acid as by‐product, which was four times less than the other isolates (data not shown). The results of 26s rDNA sequencing (Table S2) showed that the isolated yeast strain shared 100% identity with *P. kudriavzevii* strains in GenBank. The key physiological characteristics of the isolated strain are shown in Table S1. Most of the characteristics of the strain are the same as those of *P. kudriavzevii*, except for that the isolated strain can metabolize inulin and not glycerol, in contrast to previous reports of *P. kudriavzevii* (Kurtzman, 2011). Thus, the isolated yeast strain was designated as *P. kudriavzevii* SI.

### Effects of glucose concentration and temperature on ethanol production

High‐temperature fermentation process is advantageous in terms of cost reduction such as reduction in the amount of saccharification enzymes for SSF process and reduction in the cooling costs of fermentation in tropical areas (Abdel‐Banat *et al*., 2010; Hasunuma and Kondo, 2012). Furthermore, increasing the product titre in the feed to distillation greatly reduces the ethanol production costs (Sassner *et al*., 2008). Therefore, we evaluated the capacity of *P. kudriavzevii* SI for ethanol production at high incubation temperatures with high concentrations of dextrose, as shown in Table S3. When fermentation was performed with an initial glucose concentration of 87 g_s_ l^−1^ at temperatures of 37°C, 40°C and 42°C, the patterns were similar, with ethanol yields of 0.45–0.46 g_p_ g_s_^−1^ and productivities of 1.71–1.75 g_p_ l^−1^ h^−1^. At 40°C and 42°C, the strain completely utilized the glucose after only 24 h at higher concentrations of 117 and 135 g l^−1^ respectively, with ethanol yields of 0.46 and 0.47 g_p_ g_s_^−1^ and ethanol productivities of nearly 2.3 and 2.7 g_p_ l^−1^ h^−1^ respectively. However, the cells required 31 h to totally consume the glucose with concentrations of 117 and 135 g l^−1^ at 37°C. The yeast strain could also ferment at a high temperature of up to 45°C. At this temperature, the ethanol yields were 0.44 and 0.46 g_p_ g_s_^−1^, and the productivities were about 1.7 and 1.8 g_p_ l^−1 ^h^−1^, with initial glucose concentrations of 87 and 117 g l^−1^ respectively. However, the ethanol yield (0.42 g_p_ g_s_^−1^) decreased as the glucose concentration increased (134 g l^−1^). Thus, the productivity (1.85 g_p_ l^−1^ h^−1^) was 1.5‐fold lower compared with that at 40°C and 42°C (2.7 g_p_ l^−1^ h^−1^). Figure S1A and B show that 42°C was the most favourable temperature for fermentation by *P. kudriavzevii* SI. During fermentation for 10 h, the maximum ethanol productivity by strain SI at 42°C (3.93 g_p_ l^−1^ h^−1^) was higher than that at 37°C (3.47 g_p_ l^−1^ h^−1^), 40°C (3.73 g_p_ l^−1^ h^−1^) and 45°C (2.93 g_p_ l^−1^ h^−1^) when fermentation was performed with an initial glucose concentration of 87 g l^−1^. Moreover, as shown in Fig. S1C, the maximum specific growth rate of *P. kudriavzevii* SI at 42°C (0.450 h^−1^) was higher than that at 37°C (0.430 h^−1^), 40°C (0.444 h^−1^) and 45°C (0.336 h^−1^). Thus, the temperature used in the remaining fermentation studies was 42°C.

In the present study, we isolated a thermotolerant yeast *P. kudriavzevii* SI that could tolerate high temperatures ranging from 40 to 45°C, which obtained a high ethanol titre of 55.6 g_p_ l^−1^, with an ethanol yield of 0.46 g_p_ g_s_^−1^ in YPD medium at high temperatures up to 45°C.

### Influence of inhibitory compounds on ethanol fermentation by *P. kudriavzevii* SI

Several strategies have been used to overcome inhibition effects in lignocelluloses hydrolysates. Overliming is used to detoxify acid‐treated biomass (Palmqvist and Hahn‐Hägerdal, 2000). Adaptation of the microorganism in the presence of toxic compounds and genetic engineering in yeast also improved the strain's resistance to inhibitors (Jönsson *et al*., 2013). Therefore, to reduce the costs of neutralization and shorten processing time of adaptation of lignocellulose‐derived inhibitors, we evaluated the inhibitor tolerance of *P. kudriavzevii* SI by testing individual inhibitors in a synthetic medium where the initial pH was 5.0.

Increasing the concentration of any inhibitory compound decreased the glucose consumption and ethanol production rates for both *P. kudriavzevii* SI and control strain *S. cerevisiae* BCRC20270 (Fig. S2). The theoretical yield of ethanol with *P. kudriavzevii* SI was comparable to that with *S. cerevisiae* at concentration of 11 g l^−1^acetic acid. However, the maximum productivity of the isolated strain was 3.3 times higher than that by *S. cerevisiae* in the same conditions (Table 1). SI strain can tolerate up to 18 g l^−1^ of acetic acid with a 88.3% of theoretical yield, although the maximum productivity was decreased by 76.1% compared with the conditions lacking acetic acid. In contrast, fermentation by the yeast *S. cerevisiae* BCRC20270 was inhibited in the same conditions. Previous studies have proposed that the residual undissociated molecular form of acetic acid in the medium may lead to decreased fermentation rates and ethanol yields. The undissociated acid enters the yeast cell via passive diffusion across the cell membrane, and it then dissociates because of the neutral cytosolic pH, which further decreases the intracellular pH, possibly causing cell death (Zhao *et al*., 2008; Mira *et al*., 2010). The dissociation constant (p*K*_a_) of acetic acid is 4.75, and thus, the undissociated acetic acid content was 37% at pH 5.0 (Palmqvist and Hahn‐Hägerdal, 2000). Based on our results, it suggests that SI strain can tolerate at least 6.7 g l^−1^ undissociated acetic acid according to the dissociation constant of acetic acid.

**Table 1:** Comparison of the effects of lignocellulose‐derived inhibitors on fermentation by *Pichia kudriavzevii* SI and *Saccharomyces cerevisiae* BCRC20270

Inhibitor	Concentration (g l^−1^)	*P. kudriavzevii* SI			*S. cerevisiae* BCRC20270		
		*P* (g_p_ l^−1^)	*Q*_*p*_max (g_p_ l^−1^ h^−1^)	*T.Y*. (%)	*P* (g_p_ l^−1^)	*Q*_*p*_max (g_p_ l^−1^ h^−1^)	*T.Y*. (%)
Acetic acid	4	33.07 ± 0.18	2.88 ± 0.02	93.83 ± 0.64	31.55 ± 0.04	2.57 ± 0.01	89.32 ± 1.79
	7	32.37 ± 0.09	2.82 ± 0.01	92.01 ± 0.92	31.70 ± 0.10	2.36 ± 0.01	90.54 ± 1.09
	11	32.40 ± 0.14	1.67 ± 0.13	93.64 ± 1.17	30.57 ± 0.05	0.50 ± 0.01	88.35 ± 1.30
	18	30.18 ± 0.07	0.66 ± 0.01	88.29 ± 1.76	0.18 ± 0.01	0.01 ± 0.01	0.52 ± 0.01
5‐HMF	1	32.16 ± 0.03	2.01 ± 0.01	89.89 ± 0.05	31.85 ± 0.12	1.79 ± 0.02	89.54 ± 0.88
	2	30.50 ± 0.04	1.79 ± 0.13	85.73 ± 0.37	31.21 ± 0.01	0.99 ± 0.01	88.60 ± 0.42
	3	30.47 ± 0.04	1.29 ± 0.01	86.03 ± 0.24	29.90 ± 0.30	0.63 ± 0.01	85.18 ± 1.36
	4.5	29.15 ± 0.02	0.59 ± 0.01	82.97 ± 0.14	1.04 ± 0.09	0.02 ± 0.01	2.61 ± 0.33
	5	28.30 ± 0.38	0.51 ± 0.01	80.51 ± 1.25	0.70 ± 0.02	0.01 ± 0.01	2.01 ± 0.05
Furfural	1	33.54 ± 0.28	2.36 ± 0.08	87.70 ± 1.06	35.39 ± 0.71	1.88 ± 0.06	88.73 ± 2.67
	2	33.35 ± 0.18	1.96 ± 0.03	86.80 ± 0.79	34.18 ± 0.24	1.08 ± 0.01	90.77 ± 0.33
	3	33.55 ± 0.52	1.32 ± 0.02	87.85 ± 2.29	0.90 ± 0.1	0.04 ± 0.01	2.25 ± 0.22
	5	0.56 ± 0.06	0.04 ± 0.01	1.44 ± 0.76	0.60 ± 0.03	0.03 ± 0.01	1.52 ± 0.02

*P*, product (ethanol) concentration; *Q*_*p*_ max, maximum productivity of ethanol; *T.Y*., theoretical yield of ethanol.

The fermentation temperature was 42°C for *P. kudriavzevii* SI, whereas it was 30°C for *S. cerevisiae* BCRC20270.

The theoretical yield of ethanol with *P. kudriavzevii* SI was comparable to that with *S. cerevisiae* at concentrations of 3 g l^−1^ 5‐HMF and 2 g l^−1^ furfural. However, the maximum productivity of the isolated strain was 2.0 and 1.8 times higher than that by *S. cerevisiae* in the same conditions respectively. SI strain produced ethanol in the presence of inhibitor concentrations of 5 g l^−1^ 5‐HMF and 3 g l^−1^ furfural with 81% and 88% of theoretical yields respectively, whereas ethanol production by *S. cerevisiae* was inhibited in these conditions (Table 1). Palmqvist and Hahn‐Hägerdal (2000) reported that an NADH‐dependent alcohol dehydrogenase (ADH) in yeasts is responsible for the metabolism of furan derivatives such as 5‐HMF and furfural. ADH also contributes to the transformation of acetaldehyde into ethanol, so these furans will directly inhibit ADH due to substrate competition. The increased level of intracellular acetaldehyde leads to a reduced ethanol yield, longer lag phase and glycolysis inhibition in yeasts. According to our results, the mechanism that underlies furan tolerance by *P. kudriavzevii* SI remains unclear. However, it is possible that the cells synthesize more NADH, the cofactor used by enzyme ADH, compared with *S. cerevisiae*. Therefore, to reduce the amount of furan compounds and enhance biomass formation simultaneously, it might be preferable to increase the availability of the cofactor.

### Simultaneous saccharification and fermentation from AISE‐treated rice straw

**Figure 1:**
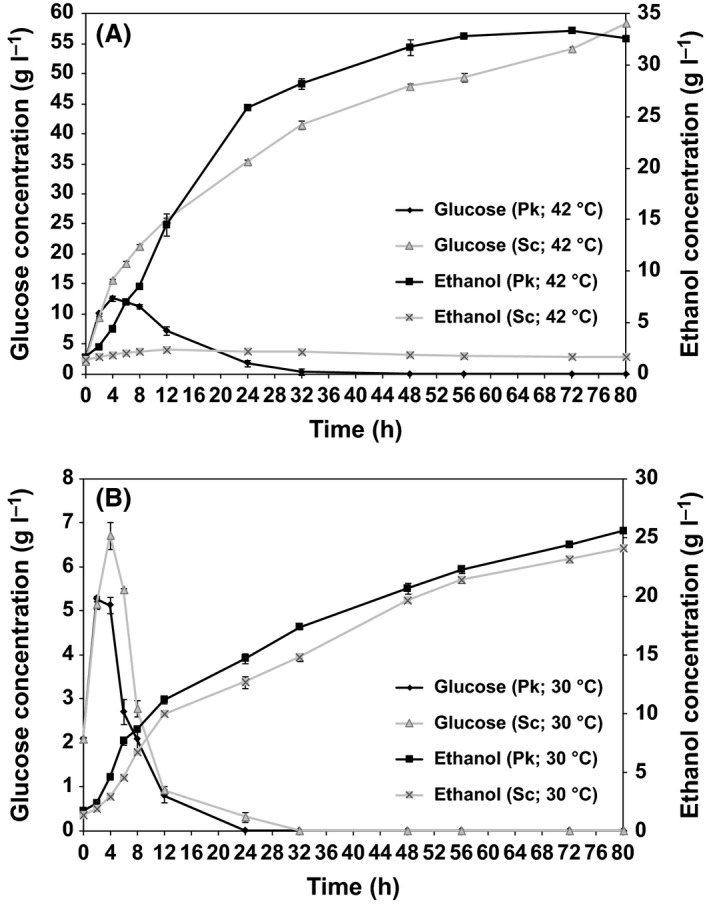
Comparison of the time‐courses for *Pichia kudriavzevii* SI and *Saccharomyces cerevisiae* during ethanol fermentation using AISE‐treated rice straw by SSF at: (A) 42°C and (B) 30°C. Sc denotes *S. cerevisiae* BCRC20270 and Pk is *P. kudriavzevii* SI.

The SSF process is more attractive than SHF due to reduced end‐product inhibition by sugars relative to cellulase, higher ethanol yields and lower investment costs for equipment (Olofsson *et al*., 2008; Hasunuma and Kondo, 2012). However, the fermentative temperature requirement for conventional ethanol‐producing yeasts normally ranges from 30 to 35°C, which is not optimal for enzymatic hydrolysis (40–50°C), and thus, there is a loss of fermentable sugar during the SSF process. To overcome the lower rate of hydrolysis as the reaction temperature decreases, we evaluated SSF process from a high solid loading of 20% (w/v) AISE‐treated rice straw with low inhibitor concentrations (furfural 0.19 g l^−1^ and acetic acid 0.95 g l^−1^) by *P. kudriavzevii* SI at 30°C and 42°C, compared with the control strain *S. cerevisiae* BCRC20270, to elucidate the fermentative capacity of the isolated strain in SSF (Fig. 1). Table 2 shows the compositions of the lignocellulosic feedstocks after pretreatment in specific operating conditions. The AISE‐treated rice straw contained 51.01% glucan (cellulose), 1.45% xylan, 0.67% arabinan and 31.60% lignin. The glucose yield in terms of enzymatic hydrolysis using the pretreated rice straw was 76.9% after 72 h (data not shown). Given its glucose content, a loading of 20% (w/v) pretreated solid residue could potentially release 87.16 g l^−1^ glucose by enzymatic hydrolysis, which was used to calculate the ethanol yield in the SSF experiments.

**Table 2:** Operating parameters for acid‐impregnated steam explosion and compositions of the pretreated lignocellulosic feedstocks

Feedstock	Raw materials				Pretreatment parameters			Pretreated materials			
		Hemicellulose									
	Cellulose Glucan (%)	Xylan (%)	Arabinan (%)	Total lignin (%)	Sulphuric acid concentration (%)	Reaction temperature (°C)	Reaction time (min)	Cellulose Glucan (%)	Hemicellulose Xylan (%)	Arabinan (%)	Total lignin (%)
Rice straw	33.1 ± 1.6	19.5 ± 1.8	4.0 ± 0.9	14.3 ± 2.9	1.5	200	1	51.01 ± 1.86	1.45 ± 0.18	0.67 ± 0.06	31.60 ± 1.00
Plywood chips	36.5 ± 0.1	23.5 ± 0.1	3.0 ± 0.0	23.1 ± 0.1	1.2	185	2	47.01 ± 0.31	0.48 ± 0.04	ND	47.49 ± 0.27
Sugarcane bagasse	44.2 ± 0.4	15.7 ± 0.1	0.8 ± 0.0	30.5 ± 0.1	1.2	185	2	51.83 ± 0.22	2.52 ± 0.36	ND	30.18 ± 0.84

ND denotes that the quantity was not detectable.

During SSF at 42°C, the maximum productivity by *P. kudriavzevii* SI within 12 h was 1.07 g_p_ l^−1^ h^−1^. The ethanol concentration at the end of 72 h was 33.4 g_p_ l^−1^ which represents 75.1% of the theoretical yield (Fig. 1A). In contrast, in the same conditions, the maximum productivity by *S. cerevisiae* BCRC20270 was 0.11 g_p_ l^−1^ h^−1^ within 6 h, and the maximum ethanol concentration was 2.36 g_p_ l^−1^, corresponding to a 2.3% of the theoretical yield. Fermentation by *S. cerevisiae* was inhibited significantly, probably due to the negative effect of high temperature on its fermentative capacity. The cascade and synergistic adverse effects of heat and cellular oxidative stressors led to decreased cell growth and ethanol production by *S. cerevisiae* at high temperatures (Aldiguier *et al*., 2004; Woo *et al*., 2014). Although SSF was performed at a lower temperature of 30°C, which is favourable for fermentation by *S. cerevisiae* cells, *P. kudriavzevii* SI exhibited a comparable fermentation capacity (Fig. 1B). The ethanol productivity by *P. kudriavzevii* SI (0.72 g_p_ l^−1^ h^−1^) was 1.8‐fold higher than that by *S. cerevisiae* (0.39 g_p_ l^−1^ h^−1^) in the first 4 h. After 6 h, the concentration of glucose accumulated by enzyme hydrolysis during fermentation was lower using *P. kudriavzevii* SI (2.7 g_s_ l^−1^) than that using *S. cerevisiae* (5.48 g_s_ l^−1^), thereby indicating that *P. kudriavzevii* SI has a more rapid glucose consumption rate than *S. cerevisiae*. At the end of 80 h, *P. kudriavzevii* SI produced about 6% more ethanol (25.6 g_p_ l^−1^) than *S. cerevisiae* (24.1 g_p_ l^−1^).

During SSF for 80 h, *P. kudriavzevii* SI produced nearly 39% more ethanol at 42°C (33.4 g_p_ l^−1^) compared with that produced by *S. cerevisiae* cells at 30°C (24.1 g_p_ l^−1^). Moreover, the maximum ethanol productivity by the isolated strain at 42°C (1.1 g_p_ l^−1^ h^−1^) was about 20% more than that by *S. cerevisiae* cells at 30°C (0.9 g_p_ l^−1^ h^−1^). These results strongly suggest that *P. kudriavzevii* SI is more suitable for practical use in ethanol production during high‐temperature SSF, and it exhibits better fermentation performance than *S. cerevisiae*. Based on the ethanol concentration and yield obtained after fermentation for 80 h, SSF at 42°C using *P. kudriavzevii* SI and SSF at 30°C with *S. cerevisiae* could produce 211 and 152 l of ethanol respectively, from one ton of AISE‐treated rice straw. Compared with the SSF process using AISE‐treated rice straw by *S. cerevisiae* at 30°C (the maximum titre is 24.1 g_p_ l^−1^ at the end of 80 h), we estimated that high‐temperature SSF at 42°C using *P. kudriavzevii* SI could shorten the fermentation time by approximately 75% to achieve a similar ethanol concentration (20 h), which could reduce the production cost. The high‐temperature SSF process using AISE‐treated rice straw in the present study was beneficial for enzyme saccharification, and the thermotolerant yeast *P. kudriavzevii* SI exhibited superior fermentation efficiency than *S. cerevisiae*.

Previously, several strains have been applied to SSF using acid‐treated lignocellulosic biomass at high temperatures ranging from 35 to 45°C, as shown in Table 3. The yeast *Kluyveromyces marxianus* has attracted much attention because of its high thermotolerance (42–45°C), and it also possesses a diverse metabolic system that can utilize a wide range of sugars (Nonklang *et al*., 2008); however, this strain has a low theoretical ethanol yield (46–65%; Ballesteros *et al*., 2008; Castro and Roberto, 2014). The zygomycete fungi *Mucor indicus* and *Rhizopus oryzae* have several industrial advantages compared with baker's yeast, such as the production of valuable chitosan and high‐efficiency ethanol fermentation at 37°C. Karimi *et al*. (2006) have studied SSF using acid‐pretreated rice straw with *M. indicus* and *R. oryzae* at 38°C, which exhibited comparable maximum productivities (0.17–0.24 g_p_ l^−1^ h^−1^) and theoretical yields (68–74%) to that obtained by thermotolerant *S. cerevisiae* Thermosacc^®^; although the yields were lower than those produced by our novel strain. Wang *et al*. (2015) have reported that fermentation using the thermotolerant *S. cerevisiae* KF‐7 at 35°C achieved a theoretical yield of 77.3%, which was comparable to our results; however, the solid loading (20% (w/v) is equal to 19.58% (w/w) derived from the density 1.12 g/cm^3^ of pretreated solid residue in dry basis) that we used and the ethanol titre (33.4 g_p_ l^−1^) with *P. kudriavzevii* SI were higher than those with *S. cerevisiae* KF‐7, that is a solid loading of 15% (w/w) and 21.5 g_p_ l^−1^ ethanol respectively. Although the concentration of acetic acid presented in their SSF process was similar with that in our study, the concentration of furans presented in their results (furfural 0.8 g l^−1^ and 5‐HMF 0.2 g l^−1^) was higher than that in our process (furfural 0.19 g l^−1^). The higher concentration of inhibitors probably resulted in the negative effect on fermentative capacity of *S. cerevisiae* KF‐7. To sum up, the maximum productivity and theoretical ethanol yield obtained from our SSF study using acid‐treated rice straw were comparable to or better than previous results.

**Table 3:** Summary of previous studies of bioethanol production from acid‐treated lignocellulosic feedstocks

Strain	Substrate	Temperature (°C)	*P* (g_p_ l^−1^)	*Q*_*p*_max (g_p_ l^−1^ h^−1^)	*T.Y*. (%)	Reference
*Kluyveromyces marxianus* NRRL Y‐6860	Dilute‐acid‐treated rice straw	45	11.55	2.70*	47.06	Castro and Roberto (2014)
*Kluyveromyces marxianus* CECT 10875	Dilute‐acid‐treated cardoon	42	23.00	–	65.00	Ballesteros *et al*. (2008)
*Saccharomyces cerevisiae* KF‐7	Dilute‐acid‐treated rice straw	35	21.50	0.90*	77.30	Wang *et al*. (2015)
*Mucor indicus* CCUG22424	Dilute‐acid‐treated rice straw	38	11.35	0.24*	67.62	Karimi *et al*. (2006)
*Rhizopus oryzae* CCUG28958	Dilute‐acid‐treated rice straw	38	12.35	0.17*	73.58	Karimi *et al*. (2006)
*Saccharomyces cerevisiae* Thermosacc^®^	Dilute‐acid‐treated rice straw	38	10.20	0.21*	60.77	Karimi *et al*. (2006)
*P. kudriavzevii* SI	AISE‐treated rice straw^a^	42	33.40	1.07	75.14^d^	This study
	AISE‐treated plywood chips^b^	42	26.31	1.96	95.48	This study
	AISE‐treated sugarcane bagasse^c^	42	22.57	0.66	91.20	This study

AISE, acid‐impregnated steam explosion; *Q*_*p*_ max, maximum productivity of ethanol; *T.Y*., theoretical yield of ethanol; *, not given directly in the referenced study and thus calculated by the authors; –, not determined in the referenced study.

Inhibitor concentrations: **a.** furfural 0.19 g l^−1^, acetic acid 0.95 g l^−1^; **b.** furfural 1.21 g l^−1^, 5‐HMF 0.72 g l^−1^, acetic acid 4.80 g l^−1^, **c.** furfural 1.07 g l^−1^, 5‐HMF 0.20 g l^−1^, acetic acid 7.65 g l^−1^, **d.** The glucose yield in terms of enzymatic hydrolysis using the pretreated rice straw was 76.9%, thereby releasing 87.16 g l^−1^ glucose; hence, the theoretical yield of ethanol was 75.14% by *P. kudriavzevii* SI.

### Separate hydrolysis and fermentation from enzyme‐saccharified hydrolysates of AISE‐treated plywood chips and sugarcane bagasse

**Figure 2:**
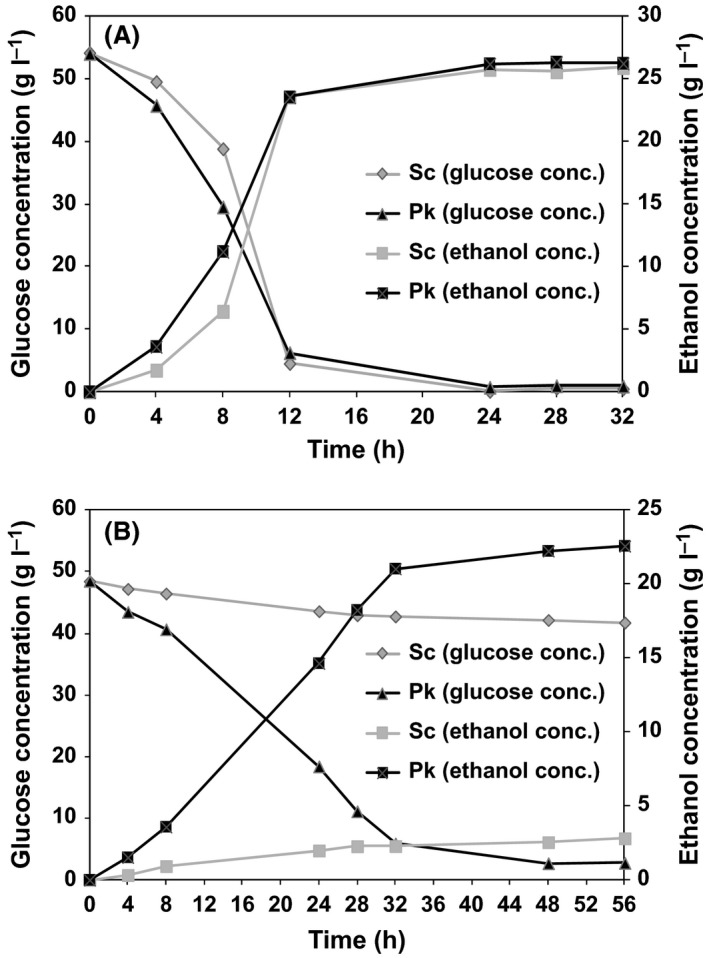
Comparison of the time‐courses for *Pichia kudriavzevii* SI and *Saccharomyces cerevisiae* during ethanol fermentation using enzymatic hydrolysate obtained from: (A) AISE‐treated plywood chips and (B) AISE‐treated sugarcane bagasse. The fermentation temperature was 42°C for *P. kudriavzevii* SI, whereas it was 30°C for *S. cerevisiae*. Sc denotes *S. cerevisiae* BCRC20270 and Pk is *P. kudriavzevii* SI.

Previous studies have demonstrated that the actual toxicity of lignocellulosic hydrolysates is attributable to the combined action of toxic compounds (alcohols, aldehydes and acids) rather than individual compounds (Nigam, 2001; Almeida *et al*., 2007). Therefore, to investigate the inhibitor tolerance of the isolated strain when using acid‐treated lignocellulosic feedstocks, we performed a comparative evaluation of ethanol production by *P. kudriavzevii* SI and the control strain *S. cerevisiae* BCRC20270 using enzyme‐saccharified hydrolysates obtained from pretreated biomass without detoxification, as shown in Table 3 and Fig. 2. The compositions of the SHF media used in this study are shown in Table S5. *P. kudriavzevii* SI produced 26.3 g_p_ l^−1^ of ethanol, which represents 95.5% of the theoretical yield, via the enzymatic saccharification of pretreated plywood chips with a high concentration of furaldehydes (furfural 1.21 g l^−1^ and 5‐HMF 0.72 g l^−1^), and the productivity (1.4 g_p_ l^−1^ h^−1^) within 8 h was 1.75‐fold higher than that by the control strain *S. cerevisiae* (0.8 g_p_ l^−1^ h^−1^; Fig. 2A). In addition, *P. kudriavzevii* SI generated 22.6 g_p_ l^−1^ of product, which represents 91.2% of the theoretical yield, via the enzymatic saccharification of pretreated sugarcane bagasse with a high concentration of acetic acid (7.65 g l^−1^). In contrast, the non‐detoxified sugarcane bagasse hydrolysate significantly hindered fermentation by the control yeast *S. cerevisiae* (Fig. 2B). These results strongly suggest that the isolate possesses greater inhibitor tolerance than the conventional ethanol‐producing strain *S. cerevisiae* when applied to pretreated enzyme‐saccharified lignocellulosic hydrolysates with high inhibitor concentrations.

Synergistic inhibition by different types of lignocellulose‐derived inhibitors has been reported for yeasts. Thus, there is a negative effect on the ethanol yield when acetic acid and furfural are present simultaneously (Palmqvist *et al*., 1999). This effect may explain why fermentation by *P. kudriavzevii* SI from the enzymatic hydrolysate of AISE‐treated sugarcane bagasse required a longer period of time than that with pretreated plywood chips in this study. This may be because of a higher synergistic inhibitory effect caused mainly by the higher concentration of acetic acid in the sugarcane bagasse hydrolysate (7.65 g l^−1^) than in the plywood chips hydrolysate (4.8 g l^−1^), despite the lower initial glucose, furfural and 5‐HMF concentrations in the sugarcane bagasse hydrolysate (48.5, 1.07 and 0.2 g l^−1^ respectively) compared with the plywood chips hydrolysate (54, 1.21 and 0.72 g l^−1^ respectively). The same phenomenon was also observed during the fermentation of AISE‐treated sugarcane bagasse using *S. cerevisiae* BCRC20270 compared with AISE‐treated plywood chips.

### Comparison of ethanol production by *P. kudriavzevii* SI and the earlier described *P. kudriavzevii* strain

Several studies have revealed the application of P. kudriavzevii to ethanol production. As shown in Table S4, the result of Yuangsaard et al. (2013) showed that P. kudriavzevii DMKU 3‐ET15 could produce 78.6 g l^−1^ of ethanol from cassava starch. Dhaliwal et al. (2011) reported that fermentation using galactose‐adapted P. kudriavzevii produced 71.9 g l^−1^ of ethanol from sugarcane juice. Although the high ethanol titres with high theoretical yields obtained from these results, in views of environmental concerns and human food chain, it is unfavourable for ethanol production from starch or food as the raw materials. P. kudriavzevii HOP‐1 has been used for ethanol production from a solid loading of 10% (w/v) alkali‐treated lignocellulosic feedstocks by SSF process (Kaur et al., 2012; Oberoi et al., 2012). HOP‐1 strain was able to produce 24.3 g l^−1^ of ethanol from alkali‐treated rice straw with 82% of the theoretical yield. The strain was also applied in fermentation from alkali‐treated cotton stalks, resulting in production of 19.5 g l^−1^ ethanol; however, the theoretical yield was merely 21%. In the present study, the solid loading (20%) that we used and the ethanol titre (33.4 g l^−1^) obtained from AISE‐treated rice straw with P. kudriavzevii SI showed a lower theoretical yield (75%) and the longer fermentation time (72 h) probably due to the higher solid loading with higher content of lignin we used results in the negative effect on the enzyme hydrolysis (Zhao et al., 2012), and without supplementary nutrients during fermentation by SI strain leads to the diminished cell growth rate as well as fermentative capacity (Broach, 2012). However, the present study is still the first report to show achievements of ethanol fermentation by P. kudriavzevii using acid‐treated rice straw.

## Conclusions

Lignocellulosic feedstocks are utilized worldwide for fuel ethanol production. In this study, we demonstrated that the novel yeast *P. kudriavzevii* SI is a robust ethanol‐producing strain that can ferment various AISE‐treated lignocellulosic biomass types without detoxification or the addition of nutrients. Compared with the conventional strain *S. cerevisiae* BCRC20270, the isolated strain has a greater capacity for high‐temperature fermentation using AISE‐treated rice straw, and it can potentially obtain 39% more ethanol in the SSF process, decreasing the possibility of bacterial contamination and reducing the cost of producing 1 l of ethanol by 0.3 US dollars in large‐scale ethanol production based on our comparison results at the conditions we ran. In addition, the inhibitor tolerance of *P. kudriavzevii* SI may eliminate the requirement for neutralization processes and the possible loss of fermentable sugars. In terms of improving the economic viability of cellulosic ethanol production, the multistress‐tolerant *P. kudriavzevii* SI may be an ideal candidate for the bioethanol industry.

## Experimental procedures

### Microorganism and culture

The *P. kudriavzevii* strain used in this study was isolated from kitchen waste by incubating at 40°C. The D1/D2 domain of 26S rDNA for sequencing was amplified using forward (5′‐AAACCAACAGGGATTGCCTCA‐3′) and reverse (5′‐TCTGCCAGCATCCGTGACCTACAC‐3′) primers. The sequence data have been deposited in GenBank under accession number KR336548. The detailed experimental procedures for isolation and identification of thermotolerant yeast strains are described in Appendix S1, Materials and Methods. *S. cerevisiae* BCRC20270 was purchased from BCRC and used in comparative fermentation studies.

### Effects of temperature, glucose concentration and inhibitors on ethanol production

The detailed experimental procedures for investigation of effects of temperature, glucose concentration and inhibitors on ethanol production are described in Appendix S1, Materials and Methods.

### Pretreatment of lignocellulosic feedstocks

Various lignocellulosic feedstocks were used in this study. Sugarcane bagasse and waste wood chips were supplied by the Taiwan Sugar Corporation (Tainan, Taiwan) and a plywood manufacturer (Sabah, Malaysia) respectively. The rice straw was collected from a field near Longtan (Taoyuan, Taiwan). Prior to pretreatment, all of the feedstocks were chopped to a size < 2 cm by a shredder and used as the raw material. Subsequently, the sliced raw materials were presoaked with dilute sulphuric acid overnight at room temperature. The solid‐to‐liquor ratio in the presoaking process was 20% w/w. The impregnated material was transferred to a 20 l vessel, which was custom fabricated as a steam explosion reactor. Saturated steam was allowed to enter the reactor, thereby heating the impregnated material to the desired temperature for a specific residence time before the reactor was suddenly depressurized. After the explosion, the resultant slurry was drained from the processor outlet and rapidly subjected to a solid/liquid separation process to obtain a cellulose‐rich solid residue for SSF and SHF (Huang, *et al*., 2011). Although we only used the cellulose‐rich solid residue of the dilute‐acid‐treated biomass for fermentation in this study, we recovered the liquid part of xylose‐rich hydrolysate from the pretreated biomass to produce other value‐added products such as xylitol or d‐lactic acid using the pentose‐fermenting strain *Candida tropicalis* JH030 (Huang *et al*., 2011) or recombinant *Lactobacillus pentosus* (data not shown).

In this study, the sulphuric acid concentration, temperature and residence time conditions during the pretreatment of different substrates were as follows: rice straw, 1.5% (w/w), 200°C, 1 min; plywood chips, 1.2% (w/w), 185°C, 2 min; and sugarcane bagasse, 1.2% (w/w), 185°C, 2 min.

### Simultaneous saccharification and fermentation of pretreated rice straw

SSF of non‐detoxified acid‐impregnated steam explosion (AISE)‐treated rice straw was performed at 200 rpm in a 5 l fermenter with a working volume of 2 l and no pH control. The solid residue from pretreated lignocellulosic slurries was mixed with deionized water, 0.25 g l^−1^ yeast solution and commercial cellulase enzyme (Novozymes Cellic^®^ CTec3). The solid‐to‐liquid ratio was 20% (w/v), the enzyme activity was 15 FPU per gram of cellulose and the pH of the medium was adjusted to 5.0 using 10 M NaOH. The glucose yield in terms of enzymatic hydrolysis for the pretreated rice straw was 76.9% after 72 h (data not shown). The temperatures used in the comparison of fermentation by *P. kudriavzevii* SI and *S. cerevisiae* BCRC20270 were both 42°C and 30°C.

### Separate hydrolysis and fermentation using enzymatic hydrolysates from the pretreated biomass

Prior to fermentation using the SHF process, enzymatic hydrolysis was performed at 200 rpm in a 5 l fermenter with a working volume of 2 l and no pH control. The solid residue from pretreated lignocellulosic slurries was mixed with deionized water and commercial cellulose (Novozymes Cellic^®^ CTec3). The solid‐to‐liquid ratio was 20% (w/v), the enzyme activity was 15 FPU per gram of cellulose and the pH of the medium was adjusted to 5.0 using 10 M NaOH. After hydrolysis for 72 h, the liquid fraction was separated from the enzyme‐hydrolysed materials by vacuum filtration and then used in the fermentation studies. Fermentation of the enzyme‐saccharified hydrolysates obtained from AISE‐treated plywood chips and sugarcane bagasse was performed in a 250‐ml Erlenmeyer flask with a working volume of 100 ml on a rotary shaker at 150 rpm. The compositions of the SHF media used in this study are shown in Table S5. The fermentation temperature was 42°C for *P. kudriavzevii* SI, whereas it was 30°C for *S. cerevisiae* BCRC20270. The initial cell concentration during ethanol fermentation was set at 0.5 g l^−1^.

### Analytical methods

The cell concentrations were measured in a 10‐mm path length cuvette using a U‐2900 double‐beam spectrophotometer (Hitachi, Pleasanton, CA, USA) at 600 nm. One unit of absorbance at 600 nm corresponded to 0.26 g l^−1^ of dry cell weight. The components of pretreated lignocellulosic feedstocks were determined according to laboratory analytical procedures recommended by the National Renewable Energy Laboratory of the U.S. Department of Energy (Sluiter *et al*., 2008). The identified components are cellulose (glucan), hemicelluloses (xylan, arabinan) and lignin.

Each fermentation sample was filtered through a 0.22‐μm filter and appropriately diluted with deionized water. The composition of the enzyme‐saccharified hydrolysate obtained from the pretreated lignocellulosic biomass included glucose, xylose, arabinose, acetic acid, 5‐HMF and furfural, which were measured by high‐performance liquid chromatography (HPLC). The quantitative analysis was performed using an Agilent 1100 HPLC system (Agilent, Palo Alto, CA, USA), which was equipped with a refractive index detector and controlled at 45°C. Separation was achieved using a Coregel‐87H3 column (Transgenomics, Apple Valley, MN, USA), which was maintained at 65°C, with 4 mM H_2_SO_4_ as the eluent and a flow rate of 0.8 ml min^−1^.

## Conflict of Interest

The authors declare that they have no conflict of interests.

## Supporting information


**Appendix S1.** Materials and Methods.
**Fig. S1.** Effects of temperature on: (A) glucose consumption, (B) ethanol production and (C) maximum specific growth rate of *P. kudriavzevii* SI.
**Fig. S2.** Influence of lignocellulosic inhibitory compounds on glucose consumption and ethanol production of: (A)–(F) *P. kudriavzevii* SI and (G)–(L) the control strain *S. cerevisiae*.
**Table S1.** Comparison of key physiological characteristics of *P. kudriavzevii* SI and *P. kudriavzevii*.
**Table S2.** The D1/D2 domain of 26S rDNA of *P. kudriavzevii* SI.
**Table S3.** Effects of the temperature and glucose concentration on ethanol production by *P. kudriavzevii* SI.
**Table S4.** Summary of previous results from published literature on ethanol production by using *P. kudriavzevii*.
**Table S5.** SHF mediums produced from pretreated lignocellulosic feedstocks.
