# Supplementary material for: Ethanol production from dilute‐acid steam exploded lignocellulosic feedstocks using an isolated multistress‐tolerant Pichia kudriavzevii strain
Source: Microb Biotechnol. 2017 May 5;10(6):1581–90. doi: 10.1111/1751-7915.12712 (PMC5658621; doi:10.1111/1751-7915.12712)
Supplement: Supplementary file 1 — Appendix S1. Materials and Methods. Fig. S1. Effects of temperature on: (A) glucose consumption, (B) ethanol production and (C) maximum specific growth rate of P. kudriavzevii SI. Fig. S2. Influence of lignocellulosic inhibitory compounds on glucose consumption and ethanol production of: (A)–(F) P. kudriavzevii SI and (G)–(L) the control strain S. cerevisiae. Table S1. Comparison of key physiological characteristics of P. kudriavzevii SI and P. kudriavzevii. Table S2. The D1/D2 domain of 26S rDNA of P. kudriavzevii SI. Table S3. Effects of the temperature and glucose concentration on ethanol production by P. kudriavzevii SI. Table S4. Summary of previous results from published literature on ethanol production by using P. kudriavzevii. Table S5. SHF mediums produced from pretreated lignocellulosic feedstocks. [file MBT2-10-1581-s001.docx]

**SUPPLEMENTAL INFORMATION FOR:**

**Ethanol production from dilute-acid steam exploded lignocellulosic feedstocks using an isolated multi-stress-tolerant *Pichia kudriavzevii* strain**

Shuo-Fu Yuan^1,2^, Gia-Luen Guo^2＊^, Wen-Song Hwang^2^

^1^Institute for Cellular and Molecular Biology, The University of Texas at Austin, Austin, Texas, USA.

^2^Chemistry Division, Institute of Nuclear Energy Research, Taoyuan, Taiwan, ROC

^＊^Corresponding author. Present address: Chemistry Division, Institute of Nuclear Energy Research, Atomic Energy Committee, Executive Yuan, No. 1000 Wenhua Rd. Jiaan Village, Longtan District, Taoyuan City 32546, Taiwan, ROC. Phone: +886 3 4711400 5122. Email: glguo@iner.gov.tw

**Inventory of Supplemental Information:**

**SI Materials and Methods.**

**Fig. S1.** Effects of temperature on: (A) glucose consumption, (B) ethanol production and (C) maximum specific growth rate of *P. kudriavzevii* SI.

**Fig. S2.** Influence of lignocellulosic inhibitory compounds on glucose consumption and ethanol production of: (A)–(F) *P. kudriavzevii* SI and (G)–(L) the control strain *S. cerevisiae*. (A) and (G) glucose consumption/acetic acid, (B) and (H) glucose consumption/5-HMF, (C) and (I) glucose consumption/furfural, (D) and (J) ethanol production/acetic acid, (E) and (K) ethanol production/5-HMF, and (F) and (L) ethanol production/furfural.

**SI Materials and Methods**

*Isolation and identification of thermotolerant yeast strains*

Kitchen waste collected from various food markets in Taiwan was placed in a 250 mL cap-free shake flask containing 20 mL culture enrichment yeast peptone dextrose (YPD) medium (10 g L^−1^ yeast extract, 20 g L^−1^ peptone, and 20 g L^−1^ dextrose) and incubated at 40°C on a rotary shaker (Orbital Shaking Incubator OSI-500R; DEAGLE, Taiwan) at 150 rpm for 2 days. A loopful of enriched culture was spread on a YPD agar plate and incubated at 40°C under micro-aerobic conditions until yeast colonies appeared. Yeast colonies were then picked and transferred to YPD broth, before cultivation at 40°C for 24 h with agitation at 150 rpm. Among the 36 strains obtained, a single promising isolate was used in this study, which we refer to as SI due to its highest theoretical yield of ethanol with the fewest byproducts (data not shown). This strain produced light cream colonies on YPD agar plates. The morphological properties of the strain were also determined. Under a light microscope, the cells on YPD agar varied from spherical to elongated in shape and they measured (1.3–6.0) × (3.3–14.0) μm. The strain SI was biochemically characterized (Table S1) and identified as *P. kudriavzevii* by the Bioresource Collection and Research Center (BCRC) in Taiwan.

*P. kudriavzevii* was first described as *Issatchenkia orientalis* in 1960 but was classified as *Pichia orientalis* in 1964 and named as *Pichia kudriavzevii* in 1965. It can be isolated from fruit and food sources (Kurtzman, 2011). Besides its application in ethanol production mentioned in the main text, the draft genome sequence of *P. kudriavzevii* M12 reveals genes coding for phytases and TY13 has been used in phytase production indicate *P. kudriavzevii* is a potential producer of phytases (Chan et al., 2012; Hellstrom et al., 2012). A high level of saturated and monounsaturated fatty acids in oil profile of MTCC 5493 strain also demonstrates its potential for biodiesel production (Santosh et al., 2013).

*Effects of temperature, glucose concentration, and inhibitors on ethanol production*

The effects of temperature and glucose concentration on fermentation were tested in a 250 mL shake flask containing 100 mL YPD medium (10 g L^−1^ yeast extract, 20 g L^−1^ peptone, and supplementary glucose concentrations of 87–135 g L^−1^), which was incubated at temperatures between 37 and 45°C with agitation at 150 rpm. The initial cell concentration was set at 0.2 g L^−1^.

The effects of inhibitory compounds were tested in 100 mL of YPD medium (10 g L^−1^ yeast extract and 20 g L^−1^ peptone, supplemented with 70 g L^−1^ glucose) with various concentrations of acetic acid (4–18 g L^−1^), 5-hydroxymethyl furfural (5-HMF) (1–5 g L^−1^), and furfural (1–5 g L^−1^), with agitation at 150 rpm. The fermentation temperature for *P. kudriavzevii* SI was 42°C, whereas it was 30°C for *S. cerevisiae* BCRC20270. The initial cell concentration for both the strains was set at 0.5 g L^−1^.

All of the fermentation experiments were performed in triplicate and samples were taken periodically for HPLC analysis. The ethanol yield coefficient (*Y_p/s_*) was calculated as the final ethanol concentration divided by the total glucose in the fermentation medium. The percentage theoretical yield (*T.Y.* (%)) was determined using the following equation: *T.Y.*(%) = (*Y_p/s_* ÷ 0.51) × 100, where 0.51 is the theoretical maximum ethanol yield per unit of glucose from the fermentation medium (g g^−1^). The specific growth rate (*μ*) was calculated using the following equation: *μ* (h^−1^) = ln(*X_2_*/*X_1_*)/(*t_2_ − t_1_*), where *X* represents the cell concentration (g L^−1^) and *t* is the fermentation time (h).

**Table S1.** Comparison of key physiological characteristics of *P. kudriavzevii* SI and *P. kudriavzevii*.

| Characteristics | *P. kudriavzevii* SI | *P. kudriavzevii* ^a^ |
| --- | --- | --- |
| Glucose | + | + |
| Inulin | + | − |
| Sucrose | − | − |
| Raffinose | − | − |
| Melibiose | − | − |
| Galactose | − | − |
| Lactose | − | − |
| Trehalose | − | − |
| Maltose | − | − |
| Melezitose | − | − |
| Methyl-a-D-glucoside | − | − |
| Cellobiose | − | − |
| Salicin | − | − |
| L-sorbose | − | − |
| L-rhamnose | − | − |
| D-xylose | − | − |
| L-arabinose | − | − |
| D-arabinose | − | − |
| D-ribose | − | − |
| Glycerol | − | + |
| Erythritol | − | − |
| D-mannitol | − | − |
| N-acethyl-D-glucosamine | + | + |
| Nitrate | − | − |

+: positive; −: negative

^a^ Data from Kurtzman (2011)

**Table S2.** The D1/D2 domain of 26S rDNA of *P. kudriavzevii* SI.

AAACCAACAGGGATTGCCTCAGTAGCGGCGAGTGAAGCGGCAAGAGCTCAGATTTGAAATCGTGCTTTGCGGCACGAGTTGTAGATTGCAGGTTGGAGTCTGTGTGGAAGGCGGTGTCCAAGTCCCTTGGAACAGGGCGCCCAGGAGGGTGAGAGCCCCGTGGGATGCCGGCGGAAGCAGTGAGGCCCTTCTGACGAGTCGAGTTGTTTGGGAATGCAGCTCCAAGCGGGTGGTAAATTCCATCTAAGGCTAAATACTGGCGAGAGACCGATAGCGAACAAGTACTGTGAAGGAAAGATGAAAAGCACTTTGAAAAGAGAGTGAAACAGCACGTGAAATTGTTGAAAGGGAAGGGTATTGCGCCCGACATGGGGATTGCGCACCGCTGCCTCTCGTGGGCGGCGCTCTGGGCTTTCCCTGGGCCAGCATCGGTTCTTGCTGCAGGAGAAGGGGTTCTGGAACGTGGCTCTTCGGAGTGTTATAGCCAGGGCCAGATGCTGCGTGCGGGGACCGAGGACTGCGGCCGTGTAGGTCACGGATGCTGGCAGA

**Table S3.** Effects of the temperature and glucose concentration on ethanol production by *P. kudriavzevii* SI

| *T*  (°C) | *S_0_*  (g_s_ L^−1^) | Time  (h) | *P*  (g_p_ L^−1^) | *Y_p/s_*  (g_p_ g_s_^−1^) | *Q_p_*  (g_p_ L^−1^ h^−1^) | *T.Y.*  (%) |
| --- | --- | --- | --- | --- | --- | --- |
| 37 | 87.48 ± 0.31 | 24 | 42.10 ± 0.29 | 0.46 ± 0.02 | 1.75 ± 0.01 | 91.05 ± 0.38 |
|  | 116.03 ± 0.60 | 31 | 55.85 ± 0.06 | 0.47 ± 0.01 | 1.80 ± 0.02 | 91.89 ± 0.62 |
|  | 135.48 ± 1.24 | 31 | 64.91 ± 0.89 | 0.47 ± 0.01 | 2.09 ± 0.03 | 91.81 ± 0.48 |
| 40 | 87.11 ± 0.16 | 24 | 41.16 ± 0.34 | 0.46 ± 0.01 | 1.72 ± 0.01 | 89.42 ± 0.91 |
|  | 117.25 ± 0.07 | 24 | 55.61 ± 0.20 | 0.46 ± 0.02 | 2.32 ± 0.01 | 90.59 ± 0.41 |
|  | 134.78 ± 0.53 | 24 | 64.84 ± 0.12 | 0.47 ± 0.01 | 2.70 ± 0.01 | 92.24 ± 0.18 |
| 42 | 87.79 ± 0.07 | 24 | 41.02 ± 0.01 | 0.45 ± 0.02 | 1.71 ± 0.05 | 88.39 ± 0.10 |
|  | 117.18 ± 0.11 | 24 | 55.35 ± 0.25 | 0.46 ± 0.03 | 2.31 ± 0.01 | 90.21 ± 0.46 |
|  | 135.38 ± 1.17 | 24 | 64.43 ± 1.00 | 0.47 ± 0.01 | 2.68 ± 0.04 | 91.22 ± 0.69 |
| 45 | 87.22 ± 1.05 | 24 | 40.49 ± 0.32 | 0.44 ± 0.01 | 1.69 ± 0.01 | 86.74 ± 0.28 |
|  | 116.55 ± 0.99 | 31 | 55.63 ± 0.24 | 0.46 ± 0.03 | 1.80 ± 0.01 | 90.38 ± 0.35 |
|  | 133.45 ± 0.71 | 31 | 57.49 ± 0.13 | 0.42 ± 0.01 | 1.85 ± 0.03 | 81.66 ± 0.22 |

*T*: temperature of fermentation; *S_0_*: initial glucose concentration; *P*: product (ethanol) concentration; *Y_p/s_*: ethanol yield coefficient; *Q_p_*: productivity of ethanol; *T.Y.*: theoretical yield of ethanol.

**Table S4.** Summary of previous results from published literature on ethanol production by using *P. kudriavzevii*.

| Strain | Solid loading ^a^ (%) | Substrate | Fermentation type/ addition of nutrients | Temperature (°C) | *P* (g L^-1^) | *T.Y.* (%) | References |
| --- | --- | --- | --- | --- | --- | --- | --- |
| *P. kudriavzevii* DMKU 3-ET15 | 25 | Cassava starch | Two-step enzymatic hydrolysis ^b^ before fermentation; 9 g L^-1^ of yeast extract, 0.5 g L^-1^ of each (NH_4_)_2_SO_4_, KH_2_PO_4_, and MgSO_4_∙7H_2_O | 40 | 78.6 | 85.4 | Yuangsaard et al, 2013 |
| Galactose adapted *P. kudriavzevii* | − | Sugarcane juice | −; 2 g L^-1^ of yeast extract, 2 g L^-1^ of peptone, and 1 g L^-1^ of MgSO_4_ | 40 | 71.9 | 85.0 | Dhaliwal et al., 2011 |
| *P. kudriavzevii* HOP-1 | 10 | Alkali-treated rice straw | SSF ^c^; 2 g L^-1^ of each yeast extract, MgSO_4_, and peptone | 40 | 24.3 | 82.0 | Oberoi et al., 2012 |
|  | 10 | Alkali-treated cotton stalks | SSF ^d^; 2 g L^-1^ of each yeast extract, MgSO_4_, and peptone | 40 | 19.5 | 21.0 | Kaur et al., 2012 |
| *P. kudriavzevii* SI | 20 | AISE ^e^-treated rice straw | SSF; without addition of nutrients | 42 | 33.4 | 75.1 | This work |
|  | 20 | AISE-treated plywood chips | SHF; without addition of nutrients | 42 | 26.3 | 95.5 | This work |
|  | 20 | AISE-treated sugarcane bagasse | SHF; without addition of nutrients | 42 | 22.6 | 91.2 | This work |

^a^ The proportion of dry solid residue in enzymatic hydrolysis.

^b^ Liquefaction step followed by saccharification step.

^C^ Enzymatic hydrolysis was performed at 50°C for 4 h to partially hydrolyze cellulose and hemicelluloses, then the reactor was brought down to 40°C after 4-h hydrolysis.

^d^ Enzymatic hydrolysis was performed at 50°C for 12 h pre-hydrolysis.

^e^ AISE: acid-impregnated steam explosion.

**Table S5.** SHF mediums produced from pretreated lignocellulosic feedstocks.

| Feedstock | plywood chips | sugarcane bagasse |
| --- | --- | --- |
| Solid loading ^a^ (%) | 20 | 20 |
| Glucose (g L^−1^) | 54.09 ± 0.09 | 48.48 ± 0.05 |
| Acetic acid (g L^−1^) | 4.80 ± 0.02 | 7.65 ± 0.04 |
| 5-HMF (g L^−1^) | 0.72 ± 0.01 | 0.20 ± 0.01 |
| Furfural (g L^−1^) | 1.21 ± 0.04 | 1.07 ± 0.03 |

^a^ The proportion of dry solid residue in enzymatic hydrolysis.

**Fig. S1.**

**(A)**

**(B)**

**(C)**

**Fig. S2.**

**(A)**

**(B)**

**(C)**

**(D)**

**(E)**

**(F)**

**(G)**

**(H)**

**(I)**

**(J)**

**(K)**

**(L)**

**Reference:**

Kurtzman, C.P. (2011) Chapter 57 - *Pichia*. In:The Yeasts: A Taxonomic Study, 5th Edition. Kurtzman, C.P., Fell, J.W., and Boekhout T. (eds). London: Elsevier. pp. 685-707.

Chan GF, Gan HM, Ling HL, Rashid NA. (2012) Genome sequence of *Pichia kudriavzevii* M12, a potential producer of bioethanol and phytase. Eukaryot Cell. Oct;11(10):1300-1.

Hellstrom AM, Almgren A, Carlsson NG, Svanberg U, Andlid TA. 2012. Degradation of phytate by *Pichia kudriavzevii* TY13 and *Hanseniaspora guilliermondii* TY14 in Tanzanian togwa. Int. J. Food Microbiol. **153**:73–77

Santosh Sankh, Meikandhan Thiru, Saurabh Saran, Vidhya Rangaswamy. (2013) Biodiesel production from a newly isolated *Pichia kudriavzevii* strain. Fuel. **106**: 690–696
